# Supplementary material for: Clinical sensitivity and time-to-result of a cascaded pooled testing approach for assessing the prevalence and intensity of Schistosoma haematobium infection
Source: PLoS Negl Trop Dis. 2024 Aug 22;18(8):e0012435. doi: 10.1371/journal.pntd.0012435 (PMC11373869; doi:10.1371/journal.pntd.0012435)
Supplement: Attachment — Submitted filename: Response letter.docx [file pntd.0012435.s003.docx]

**Dear Drs Fusco and Tamarozzi**

We would like to thank you for your comments and handling the submission and review process. We also thank very much the reviewers for their careful and thorough review and constructive comments, which have helped to improve the manuscript. We have made changes to the manuscript based on editors’ and reviewers' suggestions and described these changes in the below paragraphs. We hope that you will find our responses acceptable, and we look forward to your decision.

**A. Methods**

**Reviewer #1: (No Response)**

**Reviewer #2: The objectives are clearly articulated and the study design is sound. The population used is appropriate and the sample size is sufficient and clearly outlined. Statistical approaches seem sound (though this is not my area of expertise) and there are no ethical concerns.**

**However, as described in the general comments further analysis is required. Most importantly this relates to the performance of URS, can it be considered the preferred option of the approaches tested? An analysis of specificity of URS in this cohort should be conducted, to be able to recommend such an approach.**

**Some additional post-hoc analysis should also be conducted regarding pool sizes. The authors currently have a varied number of pool sizes with existing data, I would encourage them to conduct a sub-analysis here – this may not be possible since as I understand smaller pool sizes were only tested in case of positivity, but I encourage the authors to revisit this data nonetheless. If this is not possible, I would encourage to at least have a detailed breakdown of the positive pools, as I believe this would still be informative. For example, starting at the largest positive pool size and describing within this pool how many sub-pools were positive, until the individual positive samples are identified. This is also true for the egg counts found in each pool. These data can be included as supplementary data.**

We have conducted additional analysis as suggested and described the outputs in the result section.

- The prevalence of *S. haematobium* infection was assessed across demographic variables using urine reagent strips (URS) as a diagnostic tool (see Table 2).
- We have also conducted additional analysis for pool sizes. For each pool size, we have listed the count of pools containing *S. haematobium* eggs, the total number of pools screened for *S. haematobium* eggs, the number of pools with confirmed *S. haematobium* eggs upon testing, and the average egg count for each pool size.

**Reviewer #3:**

**Study design:**

1. **Indicate the rationale for including 5 villages from Afar and 1 village from Gambela region and give prevalence estimates for the two regions.**

We have provided explanations for the inclusion of five villages from the Afar region and one village from the Gambela region, along with presenting prevalence estimates for both regions. It reads, “*S. haematobium* is endemic in these villages in Gambela and Afar Regions, with prevalence ranging from 24.2% to 35.9% in Gambela and 5.3% to 37.0% in Afar school children [16-18]” (line 112 to 114).

2. **Indicate what data were collected (e.g. gender, age, village…)**

We have added a text explaining the demography data collected in the study. It reads, “Then children were given labeled plastic containers and demography data including age, gender and villages were collected when they brought the urine specimens.” (line 116 & 117).

3. **Describe how sample were randomly assigned to pools of 5**

We have added a text explaining the assignment procedure for the individual samples to pools of 5. It reads “In a first step, 40 plastic vials containing urine of 40 individuals were arranged randomly in 8 rows of 5 individual samples” (line 135 & 136).

4. **Indicate whether pools were prepared from samples of the same village or whether village of collection was not considered in preparing pools**

We have added a text explaining the procedures for creating the pools considering the area for sample collection. It reads “Pools were created using samples from the same region, without regard for the individual villages from which the samples were collected.” (line 144 and 145).

5. **The time to perform urine reagent strip tests was not considered in time-to-result analysis, the justification to not do so is not clear since 2 minutes per sample is a significantly relevant time**

The fact that the URS designed to provide relatively quick results, typically within minutes, was the reason for the lack of time data for testing comparison between the individual and pooled testing approaches. However, we acknowledge time comparison between the two methods when deploying URS could be important and we discussed this as a limitation of the current study. The added text reads “Moreover, the duration required for diagnosing *S. haematobium* infection using URS was not compared between IT and CPT. Such data would be beneficial in assessing how CPT could potentially save time and resources when utilizing URS for diagnosing *S. haematobium* infection.” (see line 388-391).

6. **Indicate how follow-up samples were employed**

Follow up samples were examined employing same strategy as the baseline survey. We have stated this in the revised manuscript. The added text reads “Urine sample collection, examination and pooling procedures were similar at baseline and one month after PZQ treatment.” (see line 145-147).

7. **Diagnosis of *S. haematobium* this section is entirely missing and should be integrated in the text**

We have described the methods used for the diagnosis of *S. haematobium*. The added text reads “The UFM involved filtering of urine samples through polycarbonate filter membranes (13 mm diameter and 12 to 20 μm pore size) using a syringe, followed by examining the filters under a microscope to quantify *S. haematobium* eggs [19]. The URS method entailed detecting microscopic blood (microhematuria) in urine samples, which served as a proxy for *S. haematobium* infection [19]. This was accomplished using Combur 10 Test reagent strips following the manufacturer’s instructions (see line 128-133).

8. **No description of urine filtration microscopy nor urine reagent strip is given**

We have described the UFM and URS methods used for the diagnosis of *S. haematobium*. We would like the reviewers to review our response above.

9. **No details are given on the qualification of the personnel performing tests in the field (individual testing) and in the laboratory (pooled testing) and how it might have affected the results**

We have added a text describing the qualification of the personnel who conducted the diagnosis of *S. haematobium* infection. It reads as “UFM may likely miss light infections but the microscopist who processed and examined the samples in the field and lab in the current study has experience for over a decade decreasing the chances of missing low intensity *S. haematobium* infections.” (line 166 &167).

**Statistical data analysis**

10. **Indicate that URS prevalence was also determined.**

We have explained that URS was also used to determine the prevalence of *S. haematobium* infection. The added text reads “The prevalence of infection at baseline was determined by dividing the number of individual urine samples tested positive for *S. haematobium* eggs deploying UFM or URS by an IT or CPT approach by the total number of individual samples tested.” (line 169-171).

11. **Lines 155-164, since the distribution of infection intensity (i.e. eggs count) is not normal, and non parametric tests are used for comparisons between groups, the median should be computed and reported, not the mean**

Thank you for the suggestions. Although the distribution of the egg count was not normal even after cubic, square, square root, or log transformation, due to the large size of the sample, we have reanalyzed the data applying parametric tests and updated the values in Table 1 accordingly. So, we have added the geometric mean values in Table 1, but medians were not reported.

12. **Lines 165-172, expand the description of the reference standard to make it clearer (e.g. IT+ and/or CPT+ was considered positive, IT- AND CPT- was considered negative)**

We have rephrased the text to elaborate it more. The revised text reads “In other words, an individual sample was considered positive when IT tested positive and/or CPT tested positive (i.e., IT+ and/or CPT+ was considered positive), and an individual sample was considered negative when an IT and CPT tested negative (i.e., IT- and CPT- was considered negative) (#line 182-185).

13. **Lines 173-180, clarify/simplify the description of the infection intensity (e.g. the infection intensity of the pool was estimated as the mean of infection intensity of the individual samples, the distribution of infection intensities was computed and quartiles identified, the prevalence of infection was then analyzed according to levels i.e. quartiles).**

Thank you for the suggested edits. We have revised the text. It reads, “To gain insights into the impact of infection intensity on the clinical sensitivity of a pooled examination, we assessed the change in sensitivity as a function of the mean UEC across the individual samples that made the pools. To this end, we classified the intensity into four levels. The levels were based on the quartiles (Q) of the mean UECs of individual samples across all pools of 5, 10, 20 and 40 that were tested deploying UFM (level 1: mean UECs ≤ Q1; level 2: Q1 < mean UECs ≤ Q2; level 3: Q2< mean UECs ≤ Q3; level 4: mean UECs > Q3). (line 188-193).

14. **line 175, “intensity” should be “intensity levels”**

In the revised manuscript the sentence reads as “To this end, we classified the intensity into four levels. ” (line 189)

15. **line 185,** **“processing samples and” should be deleted (otherwise the following is not clear)**

We have removed the phrase “processing samples and” from the revised manuscript (line 203).

**B. Results**

**Reviewer #1: (No Response)**

**Reviewer #2: The analysis is well outlined, executed and presented. Though it is somewhat limited and I have made some recommendations for further analysis of this valuable data set.**

Thank you. We have conducted additional analysis following the recommendations (see Table 3).

**Reviewer #3:**

1. **Table 1: include 95% CI of UFM prevalence and UFM median egg count; p-values should be columns not rows; indicate the reference group for comparison; include URS prevalence, 95% CI and p-value.**

Thank you. We have made the suggested changes in Table 1.

2. **Table 2: it would be preferable to swap columns (testing strategy) and rows (diagnostic test) so that results can be easily compared; the difference should be included before its 95% CI.**

Thank you. We have made the suggested changes in Table 2.

3. **Figure 1: in the x axis, simply indicate quartile/level 1,2,3,4; use more differentiated colors for IT and CPT**

Thank you. We have made the suggested changes in Fig 1.

4. **A justification should be given for interpreting r=0.57 as moderate correlation; an explanation should be given for the peculiar shape of the scatter plots with many data points flattened on the x or y axis.**

We have included a reference for interpreting r=0.57 as a moderate correlation (see line 201), and elucidated the reason behind the shape of the scatter plot in Fig 3. It reads, ‘There was also a moderate correlation between the UECs of individual samples estimated following the IT approach and the CPT approach (r = 0.57) (Fig 3A & Fig 3B). There was also a moderate correlation between the UECs of individual samples estimated following the IT approach and the CPT approach (correlation coefficient (r) equaled 0.57) (Fig 3A). The IT approach detected significantly more number of individual samples with S. haematobium egg which were missed by the CPT approach (points spread along either the axis). (see lines 276-280).

5. **Line 256, “when 10 ml and 15 ml…”, adding “both” would make the sentence clearer.**

With a correlation magnitude of 0.57 observed when examining both 10 mL and 15 mL volumes, we have removed the volumes to enhance the clarity of the sentence. The revised sentence reads, “There was also a moderate correlation between the UECs of individual samples estimated following the IT approach (10 mL) and the CPT approach (r = 0.57) (line 276 &277).

6. **Time to results: it would be advisable to include a table with the calculations**

**Response**: Unfortunately, we did not calculate the time taken for pooling, filtering, and examining each pool or individual sample separately. Instead, we calculated the total time for these processes for each pool or individual sample. Consequently, our data only allows us to generate estimates for total pools and total individual samples. We may not be able to conduct further analysis with enough output to be reported in a table beyond what we have already mentioned in the text.

**C. Conclusions**

**Reviewer #1: (No Response)**

**Reviewer #2:**

**1. The conclusions of the study are supported by the data presented, however further investigations and conclusions regarding the usage of URS should be added. The conclusions do not address this test method sufficiently. The public health relevance is sufficiently addressed.**

We have revised the conclusion. It includes results based on URS. The revised conclusion reads, “CPT based on UFM and URS techniques may help to rapidly estimate the intensity of S. haematobium infection and identify hotspots in a population.” (line 42 & 43; line 392 &393).

2. **A second limitation relates to the testing of the pooled samples, it seems to me that only testing 10 or 15ml of the pooled samples is a severe limitation as the contribution of the individual samples is at this point severely diluted. In my opinion, this must be stated as a limitation, with some speculation about the utility or lack thereof of repeated testing for example.**

We have addressed the examination of either 10 mL or 15 mL as a limitation of the study. The added text reads as “Second, only testing 10 mL or 15 mL could have led to falsely declaring urine samples free of S. haematobium egg due to the dilution of pooled samples. One may consider examining more volumes of pooled urine samples or decrease the number of urine samples examined per subject to reduce the dilution effect. As an illustration, in this scenario, it is anticipated that analyzing 3 x 10 mL of a pool containing 40 individual samples, rather than 10 mL, would elevate the sensitivity from 50.3% to 87.7% (= 1-(1–0.503)^3^). Obtaining large volume of urine from children may not always be feasible and could affect the time to result. Providing water to drink before urinating would help to collect good volume of urine from children.” (line 361-369).

**3. Another point of interest would be to factor in the time needed to collect sufficient samples for pooling, would this lead to an overall delay in the processing of the samples? How were the samples stored in this case?**

We have noted the possible delay in pooled testing due to the additional time required to collect a sufficient number of samples. The added text reads “Although pooling could lower cost by reducing the time for testing samples, the time needed to collect sufficient samples and resources needed for storing the samples could incur cost.” (line 386-388).

**4. Line 325: the limitations regarding sample size from individual villages should be described in more detail to highlight the specific instances where this was a limitation and why.**

We've acknowledged that the limited sample size in each village is a constraint and discussed it. It reads, “Fifth, the sample size from the various villages was inadequate to conduct robust analyses for each area individually (lines 381 & 382)”.

**Reviewer #3**

**1. Lines 288-289, the concept should be expanded and clarified: cascade pooled testing can or cannot be used for planning and evaluating mass treatment programs? if yes, how?**

Thank you. We have discussed the need for additional studies to recommend when and how to use CPT for planning and evaluating mass treatment programs. It reads, “Further studies incorporating larger urine volumes, more sensitive diagnostic tests, optimal pool sizes, and varying levels of endemicity are necessary to provide recommendations on the utilization and timing of CPT for planning and assessing mass treatment programs against *S. haematobium* infection.” (line 316-318)

**2. Lines 291-301, lower prevalence by CPT vs IT is explained with lower sensitivity at lower infection intensities. Because of a dilution effect? Should expand on that.**

We have added a text explaining the effect of dilution on the sensitivity. It reads, “The effect of dilution on sensitivity occurs because the concentration of S. haematobium eggs in a positive urine sample decreases as negative urine samples are added to the pool.” (line 300 to 302).

**3. Lines 291-301, if lower sensitivity at lower infection intensity, how a similar infection intensity estimate can be explained?**

Thank you for catching this. We have reanalyzed the data and corrected the typo on the similar infection intensity estimate between IT and CPT. The revised text reads, “the arithmetic mean log UECs of the individual samples estimated following the IT approach (mean = 0.55 eggs/10mL) was significantly greater compared to the estimate based on the CPT (mean = 0.30 eggs/10mL; p<0.001).” line 274-276.

**4. Lines 297-301, please expand the very interesting consideration on pool sizes**

We have expanded the text explaining how we would apply the findings on pool size to determine the efficient pool size. It reads, “Once the prevalence of S. haematobium infection and urine volume, along with the diagnostic test and ranges of pool sizes that maximizes the sensitivity in detecting parasite eggs are identified, online tools can be utilized to determine the most effective pool size for efficiently screening the parasite at a low cost in large-scale surveys conducted in endemic regions.” (line 329-333)

**5. Lines 308-309, it would be advisable to change in “pooled testing my help better map the distribution of high intensity infections”**

Thanks. The text has been revised as suggested. It reads, ‘…..Thus, pooled testing may help better map the diseases’ distribution of high intensity infections by rapidly screening large number of populations.” (lines 338 to 340)

**6. Lines 325-6, power calculations were not shown (line 395 in the revised manuscript)**

Our understanding was that this may not need power calculations as the size of the sample collected in some villages (Kelhat = 34; Andada = 37) was less than the size of the largest pool for the CPT (=40). So, we were not able to conduct the CPT separately for the samples collected from Kelhat and Andade.

**7. Lines 330-1, it was not clear from study design that different samples contribute different volumes of urine to the pools, clarify that point and its implications**

We have added a text explaining the contribution of each sample to the pools. It reads “As the pool size increases, the contribution of individual urine samples to the pools diminishes proportionally. For instance, with a total pool volume of 60 ml maintained across different pool sizes, each individual sample contributes 12 mL to pools of 5, 6 mL to pools of 10, 3 mL to pools of 20, and 1.5 mL to pools of 40.” (line 377-380).

**8. A justification should be given for not conducting a cost-effectiveness analysis**

We have added a text explaining the reason for not conducting a cost-effectiveness analysis in this study. It reads “Finally, cost effectiveness of pooled testing was not evaluated as the cost utilized for collecting, processing and examining of the specimens was not collected .” (line 383 & 384).

**D. Editorial and Data Presentation Modifications?**

**Reviewer #1: Some specific comments:**

**Abstract:**

**1. Why 10 and 15ml? Either the authors should explain why different volumes were used, or leave it out of the abstract.**

We have removed the phrase in parentheses which reads 10 mL and 15 mL in the abstract.

**Introduction**

**2. Line 94: the term ID has not been introduced before, please include.**

Thank you for catching this. ID was corrected as IT and defined. The revised sentence reads “we compared the clinical sensitivity and the time-to-result of an individual testing (IT) and CPT approach”. (line 94 &98).

**Methods**

**3. Line 122: why adding formalin? (line 130)**

Formalin was added to prevent the eggs of *S haematobium* from hatching (Ref. Francis J, Barrett SP, Chiodini PL. Best Practice No 174. Best practice guidelines for the examination of specimens for the diagnosis of parasitic infections in routine diagnostic laboratories. J Clin Pathol. 2003 Dec;56(12):888-91.

**4. Line 142-143: why UFM on both 10 and 15 mL in the CPT approach? Please include explanation here.**

We have explained the reason for filtering 10 mL and 15 mL for examination. The added text reads “To evaluate the performance based on the volume of urine examined and diagnostic test, CPT approach was performed by deploying UFM on 10 mL and 15 mL urine samples and URS.” (line 153 &154)

**Results**

**5. Table 1 mean UEC between regions, 2.78 in Afar and 3.05 in Gambella, the difference in mean UEC is quit small, how can this be statistically significantly different? Please check.**

**Overall, mean egg counts are relatively low, have the authors considered including the geometric mean or median for the egg count?**

Thank you so much for bringing this to our attention. We have rectified the issue with the mean egg estimates by recalculating them and updating the values in Table 1 accordingly. Additionally, we have reanalyzed the disparity in egg estimates based on demographic variables, employing a two-sample independent test for age, gender, and region, and utilizing a one-way ANOVA test for villages. We have also updated the corresponding p-values. Notably, the difference in mean egg count between children in Afar and Gambela was found to be nonsignificant (p=0.642). Furthermore, we have included the geometric mean values in Table 1.

**6. Line 239: the percentage for 15 mL should be 48.6% instead of 49.0%?**

Thank you for catching this. 49.0% corrected as 48.6%. (line 268).

**Discussion**

**7. Line 278-280, so no difference between 10 and 15mL? Also, indeed using more sensitive methods might improve the performance of pooled testing, not only molecular methods but also antigen detection methods could be highly useful (eg circulating anodic antigen).**

We have revised the text in paragraph 1 in the discussion as suggested. The revised text reads “Increasing the volume of urine (20 mL or 30 mL) and using more sensitive diagnostic methods (e.g., molecular tests, antigen detection methods) may improve the sensitivity of the CPT in detecting low intensity *S. haematobium* infections.” (line 302-304).

**8. How about those identified as positive by CPT but were initially negative by individual testing?**

Thank you. We have explained in the discussion that some individual tested positive by the CPT were negative by the IT. It reads “However, a significant number of urine samples from children identified as positive for *S. haematobium* egg by CPT were initially negative for the parasite egg by the IT.” (line 309 & 310).

**9. Line 288-289: so basically, the current CPT approach would not be recommended for planning and evaluating the success of MDA programs?**

CPT can be recommended for planning and evaluating of MDA programs but the plan should be made carefully especially in regions where the intensity of infection is low as CPT may give a biased prevalence estimate. We have revised the second paragraph to reflect this. It reads, “Prevalence data is key to decide on the initiation and scaling down or up of mass treatment programs [7]. In the current study, the prevalence of S. haematobium infection among the SAC when estimated using the CPT approach (17.4%) was lower compared to the estimate based on the IT strategy (31.3%). However, a significant number of urine samples of children identified as positive for S. haematobium egg by CPT were initially negative by the IT. This suggests that pooled testing may give biased estimates of the prevalence of S. haematobium infection at the individual level [23,24]. CPT might be used for large scale S. haematobium epidemiological surveys to rapidly identify hotspots for S. haematobium transmission, particularly where the intensity of infection is high. However, the method may increase the probability of false negative cases in areas where the intensity of infection is low. Hence, planning and evaluating the success of mass treatment programs based on CPT results should be made carefully. Further studies incorporating larger urine volumes, more sensitive diagnostic tests, optimal pool sizes, and varying levels of endemicity are necessary to provide recommendations on the utilization and timing of CPT for planning and assessing mass treatment programs against S. haematobium infection. (306-320).

**10. Line 291-292: I find it surprising that the mean UEC is the same between IC and CPT given the fact that the prevalence based on CPT was 50% lower compared to IT so more individuals negative based on CPT. What was the range in egg counts between the methods? Have the authors considered showing the geometric mean or the median egg count? Please clarify.**

Thank you for catching this. We have recalculated the mean eggs/10 mL. Indeed, the mean log UECs based on the IT approach was significantly lower than the mean log UECs based on the CPT. We have revised the results and discussion section to reflect this result.

The result section reads “The arithmetic mean log UECs of the individual samples estimated following the IT approach (mean = 0.55 eggs/10mL) was significantly greater compared to the estimate based on the CPT (mean = 0.30 eggs/10mL; p<0.001).” (line 274-276).

**11. Line 298-299: UFM is less sensitive in detecting light intensity S. haematobium infection [19]. This is repetition, it is already mentioned in the previous paragraph, and in my opinion does not need to be repeated here.**

Thank you. We have removed the sentence which reads “UFM is less sensitive in detecting light intensity *S. haematobium* infection [19]” from the discussion. (line 349)

**12. Line 322-324: unclear what is meant by ‘… pooled urine samples of volumes 10 and 15 pooled samples.’ Please clarify.**

We haver revised the sentence. It reads “As far as we are aware, this study represents the first attempt to estimate the time required for pooling urine samples and to compare the sensitivity of UFM (utilizing volumes of 10 mL and 15 mL) with URS for diagnosing S. haematobium infection in pooled urine samples.” (line 358-360).

**Reviewer #2: No modifications of existing data recommended.**

**1. In my opinion the abstract lacks clarity, particularly the ‘Methodology/Principal findings’ section. I suggest that this section is rewritten/restructured, to more clearly highlight the differences observed between the different test approaches, including segregating further the assessment of the test approaches, volume tested and time taken, as these all feature in a manner that is not immediately clear to the reader i.e. line 33 onwards would benefit from separation with a phrase such as – ‘We also compared the sensitivity of the test methods when testing 10ml vs 15ml of urine’ concluding that ‘we found this to be comparable’.**

Thank you for the suggestion. We have revised the abstract to make clear the comparison groups and results accordingly (see lines 19-45).

**2. The conclusion states that the performance of pooled testing may be compromised in endemic areas with low intensity infection, however the reason for this is not clear from the abstract.**

The conclusion that the performance of the CPT approach may fail in areas with low intensity of infection was made based on the result which showed a decreased sensitivity of the approach with a decrease in the intensity of infection “sensitivity increased significantly as a function of increasing log transformed UECs of the individual samples (OR 2.71, 95% CI 1.63―4.52).”.As the sensitivity was comparable between 10 ml vs 15ml ; and UFM vs URS, we didn’t comment/make any conclusions on the performance of the CPT approach based on the volume of urine examined or diagnostic techniques.

**3. In general, from the abstract and author summary it is not clear which test approach performed better and why.**

We would like the reviewer to see our response above stating that we have revised the abstract to make clear the comparison groups and results. We would like also to remind the reviewer that the main objective of the current study was to evaluate the performance of the cascade pooled testing in detecting the presence and estimating the intensity of S. haematobium infection using the results based on the individual testing strategy as a reference. And the study also examined whether the performance of the cascade pooled testing varies by the volume of urine examined (i.e., 10 mL vs 15 mL) and the diagnostic test (UFM vs URS). We hope the reviewer will find the revised abstract and author summary acceptable to this matter.

**4. In the background section, lines 76-78, a description of current testing approaches is needed to provide the reader with more context to the problems faced.**

Thank you. We have outlined the current testing approach used to commence and assess mass deworming initiatives. It reads “As of now, the data on the prevalence and intensity of S. haematobium infections required for the initiation or evaluation of mass treatment programs is typically gathered by individually screening subjects using urine filtration microscopy in large-scale epidemiological surveys.” (line 77-80)

**5. Line 79: ‘needed’ instead of ‘most welcome’.**

We have replaced ‘most welcome’ with “needed.”

**6. Line 85: ‘imperfect and resource-demanding’ requires explanations as to why.**

We have revised the sentence. It reads “First, we deployed urine filtration microscopy (UFM) only, which is known to be less sensitive in detecting low intensity *S. haematobium* infections”. Line 85-87.

**7. Line 94: ‘ID’ should be ‘IT’.**

We have replaced ID with “individual testing (IT)” (line 97)

**8. Line 139: delete ‘pool’ at end of line (duplicate).**

The duplicate “pool” was removed from the sentence in line 155.

**9. Line 146: illustrates – spelling mistakes.**

We do not see spelling mistakes in the word “illustrates.”

**10. Line 195: ‘included’ instead of ‘involved’.**

We have replaced ‘involved’ with “included” in line 218.

**11. Line 224: Bracket notation inconsistent as compared to lines 221 and 222.**

We have made edits to the dash symbol used in lines 245-249 to make it consistent. However, we kept the bracket notations the same to retain the meaning of the sentences.

**12. Table 2: Inclusion of raw numbers as in Table 1 would be helpful.**

We have added raw numbers in table 2.

**13. Fig 2: A description of the values that define the quartile ranges would be very helpful.**

We have added notes in fig 2 defining the intensity level. It reads “Intensity levels 1: mean UECs ≤ Q1 (25th percentile); level 2: Q1 < mean UECs ≤ Q2 (50th percentile); level 3: Q2< mean UECs ≤ Q3 (75th percentile); level 4: mean UECs > Q3”.

**14. Line 239: 49% should be 48.6%.**

49.0% is revised as 48.6% (please see line 267).

**15. Line 253: CTA is not described.**

CTA has been revised as CPT (Please see line 281).

**Reviewer #3:**

**Abstract**

**1.Line 25, UEC acronym should be introduced correctly as Urine Eggs Count, not urine samples.**

We have defined UEC as Urine Egg Count (please see line 34).

**Background**

**2. It would be advisable to expand the references to include more journal articles other than WHO reports**

We have replaced some of the WHO references in the background by peer reviewed articles.

**E. Summary and General Comments**

**Reviewer #1**:

**1.The authors present a very interesting (follow-up) study in which they determine the clinical sensitivity and time-to-result of a pooled testing approach for assessing the prevalence and intensity of Schistosoma haematobium infections. A pooling approach sounds very promising, in particular given the advantage of saving time. However, even though the pooling method is substantially faster than testing all samples individually, the overall sensitivity of the pooling method is poor, which – in my opinion – is more important than saving time. Indeed, performance can be reduced when it comes to low intensity infections as microscopy in general has limited sensitivity in such cases. However, making pools of individual urines could also have a dilution effect and further reduce the sensitivity as it might be very difficult to detect only few eggs. The authors suggest to use higher volumes of the final pools for microscopy in order to improve accuracy, but would this**

**be feasible? How would this reflect in time-to-result?**

Thank you. We have discussed the feasibility of collecting large volume of urine and its impact on time to result in the discussion. The added text reads “Obtaining a large volume of urine from children may not always be feasible and could impact the time required to obtain results. Offering water for drinking before urination could aid in collecting a sufficient volume of urine from children.” (line 367-370).

**2. What is the added value of including urine reagent strips (URS)? It might be useful for screening but will never confirm Schistosoma infection. Also, with all the different abbreviations (UFM/URS/UEC/CPT/IT) I find it rather confusing to read and understand what the authors are presenting.**

Multiple studies have suggested that urine reagent strips provide sensitivity similar to urine microscopy and have proposed them as a primary screening method, potentially supplanting urine microscopy for diagnosing *S. haematobium* in field settings in sub-Saharan Africa (SSA) (Ref 1-4). Therefore, we aimed to assess the performance of this rapid test for detecting S. haematobium in pooled urine samples. While we recognize that the use of abbreviations for testing approaches may cause confusion among readers, we opted to utilize abbreviations throughout the manuscript to avoid excessive wordiness and maintain readability.

1. Vaillant MT, Philippy F, Neven A, Barré J, Bulaev D, Olliaro PL, Utzinger J, Keiser J, Garba AT. Diagnostic tests for human Schistosoma mansoni and Schistosoma haematobium infection: a systematic review and meta-analysis. Lancet Microbe. 2024 Mar 8:S2666-5247(23)00377-4. doi: 10.1016/S2666-5247(23)00377-4.
2. Manciulli T, Marangoni D, Salas-Coronas J, Bocanegra C, Richter J, Gobbi F, Motta L, Minervini A, Bartoloni A, Zammarchi L; TropNet Schisto Task Force. Diagnosis and management of complicated urogenital schistosomiasis: a systematic review of the literature. Infection. 2023 Oct;51(5):1185-1221. doi: 10.1007/s15010-023-02060-5. Epub 2023 Jul 19. PMID: 37466786; PMCID: PMC10545601
3. Hong ST. Review of Recent Prevalence of Urogenital Schistosomiasis in Sub-Saharan Africa and Diagnostic Challenges in the Field Setting. Life (Basel). 2023 Jul 31;13(8):1670. doi: 10.3390/life13081670.
4. Chala B. Advances in Diagnosis of Schistosomiasis: Focus on Challenges and Future Approaches. Int J Gen Med. 2023 Mar 18;16:983-995. doi: 10.2147/IJGM.S391017. PMID: 36967838; PMCID:

**3. One thing the authors have investigated is the difference in sensitivity based on the number of eggs counted in the individual testing. This sounds indeed logical, but this is something that you wouldn’t be able to control in real life settings if you do a pooled testing approach, so why present results as such?**

This is indeed what we would expect, and we indeed cannot control for this but it knows gives us a quantitative understanding which infections will be missed. The intensity of infection also acts as an indicator of the endemicity level in a region or be associated with different phases of intervention programs, such as pre- versus post-treatment or control versus elimination stages. Therefore, the objective of conducting a stratified analysis on the sensitivity of pooled testing based on the intensity of individual samples comprising the pools was to identify the level of infection intensity at which pooled testing exhibits optimal performance. This information can then be utilized to make recommendations regarding the endemicity level at which pooled testing could be effectively applied for rapid diagnosis of *S. haematobium* infection without sacrificing sensitivity.

**Reviewer #2:**

**1. Degarege et al. have a presented an interesting study on an important topic. The question of appropriate diagnostic approaches is an essential one in the fight to eliminate disease such as schistosomiasis and it is commendable that they pursue this question in such detail. Additionally, they have a wealth of data and a very valuable cohort at their disposal. It is for this reason that I recommend a deeper analysis of the data to provide a more valuable resource for the community and to maximize the potential of the data at their disposal.**

**The current analyses are sound and the methods and approaches taken are all of good rationale. They provide an interesting insight into the differences between individualized and pooled testing; highlighting important considerations such as time taken, in order to recommend the best approach going forward.**

**However, what is lacking in my opinion is a true and distinct analysis and recommendation. For example, the time taken to conduct individualized testing is ~1.5x more than pooled testing. Though there is the assumption that individualized URS testing is faster than either approach and with a sensitivity of 68.8%, easily more sensitive than pooled UFM testing. Therefore, it seems that this would be the prudent approach; further comment is needed here. And importantly further analysis, as there is no commentary on the specificity of URS. To be able to recommend URS as faster screening approach (which seems the obvious outcome here) an analysis of specificity in this cohort must be conducted. This will add considerable value to the study. Some more information on the methodology used for the URS would then also be helpful.**

**This is important in order to provide further novelty, which is highlighted as a reason for pursuing this study after the authors’ initial 2015 study. URS is cited as a novelty factor in the current study but in my opinion needs to be investigated further to substantiate this claim.**

We have added results on the specificity of individual and pooled testing strategies when urine reagent strip was used for testing and discussed the results.

The added text in the result reads “UFM was 100% specific in identifying urine samples without *S. haematobium* egg through the IT and CPT approach. However, the specificity of the IT and CPT approaches in declaring urine samples without *S. haematobium* egg was 86.6% and 90.7%, respectively when URS was employed for testing. (lines 253-256).

The discussion reads “In the current study, an IT approach that employed URS detected *S. haematobium* egg in urine samples at a greater sensitivity than the CPT approach which employed UFM. An IT based on URS may allow to diagnose *S. haematobium* infection faster than the CPT approach based on UFM testing. However, URS was less specific than UFM in declaring urine samples without *S. haematobium* egg in both the individual and pooled samples. Thus, additional studies comparing URS and UFM considering different volumes of urine, sampling effort and intensity level and the time needed for URS based IT CPT needed to recommend the URS as faster screening approach for *S. haematobium* infection.” (lines 353-357).

We have also added a text describing the methods for conducting URS. It reads, “The URS method entailed detecting microscopic blood (microhematuria) in urine samples, which served as a proxy for *S. haematobium* infection [19]. This was accomplished using Combur 10 Test reagent strips following the manufacturer’s instructions. (lines 130-133).

**2. Finally, in my opinion, some additional post-hoc analysis should be conducted regarding pool sizes. In the discussion it is stated that preferred pool sizes need further investigation, so my question is why not conduct this investigation as part of this paper? This would add considerable value here. The authors currently have a varied number of pool sizes with existing data, I would encourage them to conduct a sub-analysis here if possible – this may not be possible since as I understand smaller pool sizes were only tested in case of positivity, but I encourage the authors to revisit this data nonetheless. If this is not possible, I would encourage to at least have a detailed breakdown of the positive pools, as I believe this would still be informative. For example, starting at the largest positive pool size and describing within this pool how many sub-pools were positive, until the individual positive samples are identified. This is also true for the egg counts found in each pool. These data can be included as supplementary data.**

We have conducted additional analysis as suggested and described the outputs in the result section. Please see table 3.

Table 3. Sensitivity of the cascaded pooled testing in detecting Schistosoma eggs in pooled urine samples

| Pool size | Total number of pools | Number of pools with *S. haematobium* egg * | Number of pools checked for *S. haematobium* (remaining pools assumed negative for Sh egg without testing) | Number of pools with *S. haematobium* egg upon testing (sensitivity) | | | Artihemitic log mean UEC of positive pools per 10 mL urine |
| --- | --- | --- | --- | --- | --- | --- | --- |
|  |  |  |  | UFM 10 mL | UFM 15 mL | URS |  |
| 40 | 34 | 33 | 34 | 24  (72.7) | 23  (69.7) | 21  (63.6) | 1.43 |
| 20 | 69 | 64 | 48 | 33  (51.6) | 31  (48.4) | 35  (54.7) | 1.49 |
| 10 | 141 | 126 | 68 | 72  (57.1) | 70 (55.6) | 75  (59.6) | 1.54 |
| 5 | 283 | 216 | 142 | 66 (30.6) | 68  (31.5) | 90  (41.7) | 8.35 |
| Number of pools with *S. haematobium* egg *: Number of pools with the mean UEC of individual samples making them different from zero and/or pools tested positive for *S. haematobium* egg via UFM per 10 mL used as a reference to calculate the sensitivity of the CPT | | | | | | | |

**Reviewer #3**:

**1. The manuscript by Degarage and colleagues aims at comparing Schistosoma haematobium cascade pooling testing vs individual testing in terms of i) prevalence of infection ii) intensity of infection iii) time-to-result. The study was conducted among school-aged children in villages from Afar and Gambella regions in Ethiopia, and employed parasitological (urine filtration microscopy) and lateral-flow (urine reagent strips) assays for diagnosis. The results are of relevance for the epidemiology and control of S. haematobium infection in Sub Saharan Africa: while cascade pooled testing lacks the necessary sensitivity needed for estimating population infection prevalence, it might be a faster method to detect transmission hotspots and estimate population infection intensity. However, the originality is limited since both the authors and others have previously provided similar evidence, cost-effective analysis is not conducted, and real life cases for applications are**

**not discussed. Also, methodology lacks some important details, and reporting and discussion of the results needs to be improved for better clarity.**

Thank you for the feedback. We have made revisions on the methodology, results and discussion sections to improve clarity on the study designs, findings and applications. However, the current study is unique compared to the previous proof of principle study in the following aspects as described in the background (see lines ## 88-97)). The main difference between the current and the previous proof of principle study is summarized in the table below.

|  | Previous study | Current study |
| --- | --- | --- |
| Regions where samples obtained from | Afar | Afar and Gambela |
| Methods used for checking S. haematobium eggs in pooled samples (i.e., performance of pooled testing was evaluated for…) | Urine filtration microscopy (only 10 mL urine examined) | Urine filtration microscopy  (10 mL and 15 mL urine examined )  Urine reagent strips |
| Largest pool size examined | 20 | 40 |
| Prevalence at the individual level determined based on the group test | No | Yes |
| Time to results data | No | Yes |
