# Supplementary material for: Clinical sensitivity and time-to-result of a cascaded pooled testing approach for assessing the prevalence and intensity of Schistosoma haematobium infection
Source: PLoS Negl Trop Dis. 2024 Aug 22;18(8):e0012435. doi: 10.1371/journal.pntd.0012435 (PMC11373869; doi:10.1371/journal.pntd.0012435)
Supplement: Attachment — Submitted filename: Response to reviewers comments.docx [file pntd.0012435.s004.docx]

**Dear Drs Fusco and Tamarozzi**

We would like to thank you for your comments and handling the submission and review process. We also thank very much the reviewers for their careful and thorough review and constructive comments, which have helped to improve the manuscript. We have made changes to the manuscript based on editors’ and reviewers' suggestions and described these changes in the below paragraphs. We hope you will find our responses acceptable and look forward to your decision.

**A Methods**

**Reviewer #1: (No Response)**

**Reviewer #2:**

1. One limitation is the lack of a specific reference test, e.g., PCR testing. The gold standard used here is a composite of IT and CPT, however, I think it is a limitation that there is no independent test method used as a gold standard and this can be stated as such.

**Response**: Thanks. We have discussed the lack of a specific reference test as a limitation. It reads, “ Sixth, this study did not employ a highly sensitive independent gold standard test (e.g., PCR) as a reference to determine the sensitivity of CPT using UFM or URS.” (lines 479& 480).

2. I also suggest a more comprehensive overview of existing test methods be included in the introduction around line 87.

**Response**: Thanks. In the introduction, we reviewed various methods available for testing S. haematobium. The added/revised text reads as follows:

“First, we deployed UFM only, which is known to be less sensitive in detecting light S. haematobium infections [5]. Other methods that involve the detection of S. haematobium DNA (e.g., PCR, loop-mediated isothermal amplification (LAMP)), antigen (e.g., point-of-care circulating cathodic antigen (POC-CCA)), or antibody released by the body against the parasite (e.g., ELISA, Indirect Hemagglutination Assay (IHA)) are available to diagnose S. haematobium infection [5]. Each method has issues with accuracy, efficiency, or accessibility [5]. Alternative cheap point-of-care tests are urine reagent strips (URS).” (lines 88-93).

**Reviewer #3**

3. The aim of the study is to investigate whether cascade pool testing might be a cost-effective alternative to individual testing for estimating the prevalence and intensity of S. haematobium at population level, by comparing CPT and IT performed by urine filtration microscopy or urine reagent strip, and by using 10ml or 15ml of urine volume.The study design is not entirely appropriate to address the aim and objectives since time-to-result analysis is limited and cost-effectiveness analysis was not conducted.

**Response**: Thanks for the comment. As described in the abstract and introduction, this study aimed to compare the clinical sensitivity and the time-to-result of individual testing (IT) and CPT approach for assessing the prevalence and intensity of *S. haematobium* infection. However, we acknowledge that the data/results on time may not be detailed enough and have conducted retrospective analyses comparing the time to results between the IT and CPT when URS was used for testing. We have updated the abstract (lines 41 & 42), methods (lines 172-176) and the results (lines 384-387) accordingly. We also agree that the cost data will be super important in evaluating whether pooling is a cost-saving strategy for diagnosing *S. haematobium* infection. Unfortunately, we didn’t collect cost data. So, we have discussed this as a limitation of the study. It reads, “Finally, cost-effectiveness of pooled testing was not evaluated as the cost utilized for collecting, processing and examining the specimens was not collected. A cost-benefit analysis or simulation study would help to verify a cost-effective large-scale survey based on pooled testing for the control of S. haematobium infection [28]. Although pooling could lower cost by reducing the time for testing samples, the time needed to collect sufficient samples and resources needed for storing the samples could incur cost.” (lines 482-486).

Moreover, to avoid misinterpretation, we have revised the text in the methods which reads “To verify whether our CPT is a cost-saving strategy,…..” as “To verify whether our CPT saves time for testing……..,”

4. Power analysis was not conducted and the sample size was considered insufficient to conduct the analysis at village level.

**Response**: we have conducted sample size/power analysis and described the procedures in the methods. The added text reads as follows:

“The prevalence of *S. haematobium* infection was 20% in Afar and 35.9% in Gambela [17,18]. A previous study showed that UFM based pooled testing (based on pools of 10 samples) is 68.6% sensitive to determining the prevalence of *S. haematobium* infection in a population [14]. Hence, to accurately estimate the sensitivity of the pooled testing in determining the prevalence of *S. haematobium* infection with 80% power and 95% CI, we need a minimum sample of 708 in the Afar and 410 in the Gambela [20] (lines 202-207).”

We have discussed the small size of the samples that prohibit analysis at the village level as a limitation of the study. It reads as follows: “Third, the sample size collected in some villages was insufficient to analyze the data at the village level with sufficient power.(lines 465 and 466)”

5. Statistical methods should be carefully revised. Variation of prevalence and intensity of S.haematobium infection with demographic factors should be evaluated by multivariate analysis. Egg counts should be analysed by non-parametric tests. Egg counts should be analysed among positive samples only.Other specific suggestions are given below.

**Response**:

i. **Multivariate analysis**: We have conducted additional analysis to examine sociodemographic factors predicting the presence and intensity of S. haematobium infection and updated the methods and results sections. The added text in the methods reads “To further explore sources of variation in the prevalence and intensity (UEC) of *S. haematobium* infection, we built Generalized Linear Models (GLM) with the test result (positive vs. negative and log urine egg count, UEC) as outcome variables. We used age group (5-9 years *vs*. 10-15 years), sex (females vs. males) and villages (6 villages) as fixed effects and the region where children live as a random variable (lines 219-224).

The added text in the results section reads as follows:

“ In the GLM that included age group, sex and villages as fixed effects and region as a random variable, the odds of S. haematobium infection was significantly lower in children who live in villages of the Afar region (Kusra aOR: 0.54, 95% CI: 0.39, 0.76; Hassoba aOR: 0.03, 95% CI: 0.010, 0.099; Kelhat aOR: 0.33 95% CI: 0.14, 0.77; Andada aOR: 0.16; 95% CI: 0.05, 0.45) compared to those who live in the Abobo village in Gambela region. The log UECs were also significantly lower in children who live in villages of the Afar region (Kusra β: -0.46, 95% CI: -0.74, -0.18; Hassoba β: -4.94, 95% CI: -7.30, -2.57; Kelhat β: -1.19 95% CI: -2.01, -0.37; Andada β: -1.52; 95% CI: -2.41, -0.63) than those in the Abobo village in Gambela region. The log UEC was lower in males than females (β: 0.30 95% CI: 0.09, 0.52). However, the differences in the prevalence and intensity (log UEC) of infection was not significant between males and females. (lines 270-280)

ii) Egg counts should be analysed by non-parametric tests: we have compared differences in egg count across sociodemographic variables deploying nonparametric tests and updated the p-values in table 1 accordingly. The added text in the methods reads as follows: “As the distribution of the egg count was not normal even after cubic, square, square root, or log transformation, two-sample Wilcoxon rank-sum (Mann-Whitney) test was used to compare mean egg count by age groups, gender and the region where children live, and the Kruskal-Wallis test was used to compare mean egg count by villages of the children (line 216 to 220).”

iii) Egg counts should be analysed among positive samples only. We have estimated the egg count after excluding individuals with zero egg count.. We have updated the data in Table 1 accordingly.

**B Results**

**Reviewer #1: (No Response)**

**Reviewer #2:**

6. The authors made an effort to include further analysis and the inclusion of Table 3 is noted. However more description of this table in the results section is required. Particularly explaining the rationale as to why not all positive pools were then tested.

**Response**:

As described in the methods section (line 156-163, figure 1B) and presented in Table 3, all the smaller pools that made the higher pools were tested when the corresponding higher pools were tested positive.

We have added more text describing the results in Table 3. The added text in the results section reads as follows:

“Table 3 summarizes the sensitivity of the cascaded pooled testing in detecting *S. haematobium* eggs in pooled urine samples using UFM and URS techniques. All 34 pools of 40 samples were tested, and 24 (72.7%) were found positive when 10 ml urine was examined using UFM. As a result, the corresponding 48 pools of 20 samples (out of 64 pools of 20 samples positive with S. haematobium egg and/or with non-zero mean UEC of individual samples), which made the 24 positive pools of 40 samples, were tested, and 38 (59.4%) were found positive when 10 ml urine was examined using UFM. Consequently, 76 pools of 10 samples (out of 129 pools of 10 samples positive with S. haematobium egg and/or with non-zero mean UEC of individual samples), representing the 38 positive pools of 20 samples, were examined, with 75 (58.1%) testing positive when 10 ml urine was examined using UFM. Then, the corresponding 150 (out of 223 pools of 5 samples positive with S. haematobium egg and/or with non-zero mean UEC of individual samples) pools of 5 samples corresponding to the 75 positive pools of 10 samples were tested, and 109 (48.9%) were found positive when 10 ml urine was examined using UFM.

Overall, the clinical sensitivity of the UFM based CPT in detecting S. haematobium eggs in pooled urine samples tends to increase with an increase in pool size (Chi2 for trend =7.12, P = 0.008 when 10 ml urine was examined using UFM; Chi2 for trend =5.65, P = 0.017 when 15 ml urine was examined using UFM). However, the sensitivity of CPT in detecting S. haematobium eggs in pooled urine samples was comparable when results were compared based on diagnostic test (UFM vs. URS) or the volume of urine examined using UFM (10 mL vs. 15 mL) (Table 3)).” (Lines 339-357).

7. Furthermore, specificity values as referred to in line 374 should be conducted or included as a Table/Figure. Including at the minimum specificity values but possibly also negative and positive predictive values would be helpful.

**Response**: We have added results on the specificity and predictive values of the test (line 282-294). The added text reads as follows: “UFM was 100% specific in identifying urine samples without S. haematobium egg through the IT and CPT approach. However, the specificity of the IT and CPT approaches in declaring urine samples without S. haematobium egg was 86.9% and 91.1%, respectively when URS was employed for testing.

The proportion of urine samples tested positive for *S. haematobium* egg using UFM that were true positive was 100% through the IT and CPT approach when the volume of urine examined was 10 ml. The proportion of urine samples tested negative for *S. haematobium* egg using UFM that were true negative was 97.2% through the IT; 80.5% by the CPT when the volume of urine examined was 10 ml and 80.2% when the volume of urine examined was 15 ml. However, the positive predictive values of URS were 73.0% by the IT and 74.5% by the CPT. The negative predictive values of URS were 85.2% by the IT and 78.9 % by the CPT.” (lines 318-329).

**Reviewer #3:**

8. The results are still not clearly presented. The tables needs careful revision and integration. The quality of the immages should also be improved. Specific suggestions are given below.

Response: We have revised the data in the Tables and the text in the results section. We have also made edits on the figures to improve their quality.

**C. Conclusions**

**Reviewer #1**: (No Response)

**Reviewer #2**: The authors provide a solid overview of the limitations of this method, it seems clear that the sensitivity of such an approach is lacking, however, it is a topic which merits investigation and so it is beneficial to know the best approaches to explore and those to avoid.

9. Line 329 - An alternative argument is that IT UFM suffers from a lack of sensitivity, which is true particularly when testing samples once. Here this could instead argue for repeated testing of the same samples or participants.

**Response**: We have recommended the evaluation of repeated testing of the same samples or participants to improve the sensitivity of pooled testing in future studies. It reads, “Further studies incorporating larger urine volumes, more sensitive diagnostic tests, repeated testing of the same samples or participants, optimal pool sizes, and varying levels of endemicity are necessary to provide recommendations on the utilization and timing of CPT for planning and assessing mass treatment programs against S. haematobium infection (lines 410-414).

**Reviewer #3**:

10. The authors discuss strenghts and limitations of their results in view of the literature. However, the significance of the study and the advancement in the topic under study are limited by issues with methodology and results presentation.

**Response**: As described in our response to the comments above, we have made significant revisions in the methods and results sections. We hope this will address it.

**D**. **Editorial and Data Presentation Modifications?**

**Reviewer #1**: (No Response)

**Reviewer #2**:

11. Line 339 - include figure reference.

**Response**: Fig 3 was cited at the end of the sentence: "The scatter plot showed positive trends in the relationship of the mean UECs of the individual urine samples estimated based on the IT and the UECs estimated using the CPT (Fig 3)”. (see line 417 & 418)

12. Spelling and grammar mistakes e.g. line 373 - should be 'diagnosis of'

**Response**: We have edited the manuscript for spelling and grammar errors. The word “diagnsose” has been corrected as “diagnose” in line 451. The sentence reads “URS may allow to diagnose *S. haematobium* infection……”

**Reviewer #3**:

13. It is still not clear why cost-effectiveness was not evaluated despite its importance is stressed in the introduction (“However, these surveys are resource demanding, and more cost-effective strategies are needed. A cost-saving alternative is a pooled sample testing approach” and “it remains unclear whether such a CPT would reduce the time-to-result, and therefore can be recommended as a cost-efficient alternative to the current diagnostic approach”), despite such evaluation is stated as one aim of the study in the methods (“To verify whether our CPT is a cost-saving strategy”) and despite it could be estimated even retrospectively

**Response**: We acknowledge that cost-effectiveness data could be valuable in recommending pooled testing for determining the prevalence and intensity of infection. Unfortunately, cost data was not collected in this study and has been discussed as a limitation; “Finally, cost effectiveness of pooled testing was not evaluated as the cost utilized for collecting, processing and examining of the specimens was not collected. A cost-benefit analysis or simulation study would help to verify a cost-effective large-scale survey based on pooled testing for the control of *S. haematobium* infection [28]” Although pooling could lower cost by reducing the time for testing samples, the time needed to collect sufficient samples and resources needed for storing the samples could incur cost. (line 480 to 486)”. We have also edited the text in the methods sections as “ To verify whether our CPT saves time…..”

14. Is it still not clear why time-to-result analysis includes UFM but not URS performed on pools vs individual spercimens, this could be done even retrospectively since URS should take a standard amount of time per sample

**Response**: We have calculated the time to test samples using the URS through the IT and CPT and updated the methods and results sections. It reads as follows:

Methods: As the time spent for processing samples and checking *S. haematobium* eggs using URS was not collected during baseline data collection, time data for the IT and CPT using URS was estimated retrospectively. Testing for *S. haematobium* infection using the URS involves dipping the strip in urine, waiting for a period, and then reading the results. This process takes approximately 2 minutes (lines 172-176).

Results : A total of 46.9 hrs was needed to check *S. haematobium* infection from 1,406 urine samples through the IT approach by URS, but the time needed decreased to 33.9 hrs (27.7%) when the CPT was applied (lines 380-382).

15. It is still not clear how data were collected: there is no mention of data collection forms and data management procedures.

**Response**: thanks. Detailed note about data collection form and management procedures has been added. It reads as follows (line 179-201):

**Data collection form and management process**

In this study, a carefully designed form was used to ensure the collection of accurate data points directly relevant to the study's objectives including demography, which consists of age in years, gender, region and village names where the children live, and lab data including *S. haematobium* egg counted through UFM technique and results about the presence of blood in urine through URS. The form was structured to provide unambiguous questions and collect quantitative and qualitative data. To ensure consistency and easier comparison and aggregation of data across different villages and regions, standardized gradings/units were used to record the test results of URS and UFM. The URS test results were documented as zero (negative), ± (trace), + (weak), ++ (moderate), and +++ (strong) erythrocytes per microliter of urine. The UFM results were recorded as the number of *S. haematobium* eggs counted in 10 ml (IT and CPT approaches) or 15 ml urine (CPT approach).

Robust data management procedures were implemented to ensure the integrity, confidentiality, and accessibility of the data. In the field, data were collected on paper forms and manually entered into a secure electronic database after the lab examination was completed. Once one person entered the data, a second person cross-checked it, and discrepancies were resolved through discussion. After removing personally identifiable information, all data were stored in a secure, password-protected local hard drive, with access restricted to authorized personnel only. Manual reviews were conducted before analysis to identify and correct inconsistencies, missing values, and outliers to ensure data quality. The data were deposited in reputable data repositories for long-term preservation and accessibility. Metadata, including descriptions of variables and coding schemes, was provided to the PLOS NTD journal to enable other researchers to understand and utilize the data effectively and reproduce the results” (lines 178-200).

16. It is still not clear how the samples were randomised for grouping and pooling: there is no mention of randomization procedures.

**Response**: We have revised the pooling procedure to make clear on the randomization

procedure. The revised text read as follows: “In a first step, we determined the number of pools of 40 samples to be made at the village (when enough sample size) or region level. Then, 40 plastic vials containing urine of 40 individuals were arranged in increasing order of their unique identifier number (ID) in 8 rows/groups of 5 individual samples. Assuming no link exists between individual ID and egg count, we considered the samples to be arranged randomly (lines 140 to 145) .

17. Why parametric tests were used to compare the distribution of eggs number between CPT and IT, given the distribution is not normal? In the previous version of the manuscript, non parametric tests were correctly used. In the point-to-point answers to reviewers comments, it is stated that parametric tests can be used since the sample size is large (?), but no justification is given for not using non-parametric tests. Also, in some part of the manuscript a geometric mean is reported, in others the arithmetic mean. I reckon that boxplot should be used instead of bars to show distribution of egg counts and that non-parametric tests should be used for comparison. Also, the comparison in distribution of egg counts and their correlation should be tested among positive samples only. The results and interpretation have changed from the previous version of the manuscript. I strongly suggest a thourough statistical revision of the manuscript.

**Response**: Because the sample size was large and the mean UEC represented the distribution of the UEC better than the median, we applied parameteric test which could powerfully/accurately detect the difference rather than non parameteric tests. However, per your recommendation, we have reanalyzed the data using non parametic test (e.g. two-sample Wilcoxon rank-sum (Mann-Whitney) test; Kruskal-Wallis test; Generalized linear model) to compare mean UEC across demographic variables. Mean egg counts were calculated among positive cases only. Moreover, we have applied a genralized linear model to examine demographic correlates of infection. Furthermore, we have conducted chisqure for trend analysis to evaluate the changes in sensitivity at the group level across pool sizes. We have described the details of the changes in the above comment. However, we think that the bars would help better convey all the information about the intensity level (light vs heavy), intensity quartiles (1, 2, 3 and 4), and sensitivity data all at once than the box plots. So, we kept the bars. In addition, pools were made by randomly mixing individual samples with positive or negarive results. There were four different pool sizes and a given pool sample could have more than one positive individual samples. Thus, limiting the correlation analysis between UEC of individual samples will be challenging, making the pools and UEC of the corresponding pools jut to positive ones only. Moreover, one of the reviewers suggested to report the geometric mean egg counts in Table 1 in addition to the arithmetic mean values. So, we have added geometric egg counts in Table 1.

18. The comparison of prevalence and intensity of S. haematobium infection according to demographic factors (Table 1) should be performed by multivariate analysis

**Response**: As described above, in addition to the Wilcoxon rank-sum (Mann-Whitney) test and the Kruskal-Wallis test, we have used generalized linear models that included age, gender, village and region to examine demographic correlates with the prevalence of of S. haematobium infection.

19. The title of the first results paragraph and the title of Table 1 should be “Prevalence and intensity of…”

**Response**: Thanks. Revised as suggested.

20. Table 1 column 3 should be “Percentage of IT/CPT positive samples by UFM (95% CI)”

**Response**: Thanks. Revised as suggested.

21. Table 1 column 4 should be “Egg count (geometric mean) by UFM”

**Response**: Thanks. Revised as suggested.

22. Table 1 column 5 should be “Percentage of IT/CPT positive samples by URS (95 % CI)”

**Response**:Thanks. Revised as suggested.

23. In Table 1 and Table 2 the 95% CI of the proportion would be better indicated by the lower confidence limit – upper confidence limit (use dash instead of comma)

**Response**: Thanks. Revised as suggested.

24. In Table 1, p-values would be better reported in a column not a row, since it is not a category of the variables (this was already suggested in the first review)

**Response**: Thanks. Revised as suggested.

25. In Table 2 column 1, Urine Filtration Microscopy should be used instead of microscope

**Response**: Thanks. Revised as suggested.

26. In Table 2 column 5, change title to “Difference between IT and CPT when using 10 ml samples (95% CI)

**Response**: Thanks. Revised as suggested.

27. In Table 2, the p-value for the difference should be reported

**Response**: Thanks. P-values for the difference added in Table 2.

28. “UFM was 100% specific in identifying urine samples without S. haematobium egg through the IT and CPT approach”: this was an assumption on which the standard reference for comparing IT and CPT was built, therefore cannot be a result

**Response**: We agree with the suggestion not to report specificity values as results. However, the second reviewer suggested to do so including predictive values in the results section (please see comment # 7). So, we reported the specificity and predictive values in the results sections. Indeed, the data confirmed 100% specificity and negative predictive values. We defer the decision to the editors on whether we should keep the specificity and predictive value data in the results section.

29. Table 3 is not at all clear and is not described either by a legend or in the text. What analysis was conducted, to what aim? Why not all pools were tested? Why some pools were assumed to be negative?

**Response**:

Table 3 is not at all clear and is not described neither by a legend or in the text: We have now added details describing the data in Table 3.(please see lines 341-359)

What analysis was conducted, to what aim?: We have performed a chi-square for trend analysis to examine the linear trend of sensitivity of the CPT with an increase in pool sizes from 5, 10, 20 to 40. However, most of the data in the table is a descriptive analysis of percent/sensitivity values. The results summarize data on the sensitivity of the CPT in detecting *S. haematobium* infection in pooled samples and whether this sensitivity vary by diagnostic test, pool size and volume of urine examined.

Why not all pools were tested? Why were some pools assumed to be negative?: As described in the methods section ( see lines 156 to 163 and Fig 1 B), the corresponding lower pools making higher pools were tested when the higher pool tested positive. When the higher pool tested negative, the corresponding lower pools were assumed negative without testing. This is how pooled sample testing strategy expected to save resources and time for testing.

30. Figure 3: correlation between egg counts should be performed on positive samples only. The sensitivity, i.e. ability to detect positive samples, was already compared.

**Response**: Thank you for your suggestion. However, there were four different pool sizes and each pools were made by randomly mixing individual samples with positive or negarive results. Each pools could contain different number of individual samples with S. haematobium egg. Thus, it will be challenging to limit the correlation analysis to just positive ones.

**31**.Time-to-result data and results paragraph is extremely limited, and it seems insufficient given

the importance of this aspect in the significance of the study.

**Response**: We have conducted additional analysis comparing the time to results between IT and CPT approaches when URS was employed to test and expand the data and text in the results section (lines 379-386).

32.“The scatter plot showed positive trends in the relationship of the mean UECs of the individual urine samples estimated based on the IT and the UECs estimated using the CPT. There was also a moderate correlation in UEC estimated by the IT and CPT approach” The two sentences are redundant, please rephrase.

**Response**: We have removed the sentence which reads “There was also a moderate correlation in UEC estimated by the IT and CPT approach.”

33. The clarity of the discussion could benefit from being restructred so that first results are summarised and interpreted, then limitations are discussed, and finally their significance is described in the light of the existing literature and of the study aims.

**Response**: We have made some revisions to the discussion. We have provided a summary of the main findings in the first paragraph. Then, we have interpreted these findings with literature discussed their implications and limitations. Finally, we have made a conclusion and made some recommendations.

**D. Summary and General Comments**

**Reviewer #1**

31. The authors have correctly addressed all my comments and questions and I am satisfied with the changes made in the manuscript. The revised manuscript is acceptable for publication.

**Response**: Thank you for the careful and thorough review and constructive comments, which have helped to improve the manuscript. I very much appreciate your time and commitment to improve the paper.

**Reviewer #2**

32. The authors have made a comprehensive effort to address the comments on the initial manuscript, and I believe the current manuscript sufficiently highlights the limitations of this approach, while still indicating that this is an important topic that requires further study.

**Response**: Thank you for the careful and thorough review and constructive comments on the first and second version, which have helped to significantly improve the manuscript. I very much appreciate your time and commitment to improve the paper.

**Reviewer #3**:

33. The overall clarity of the manuscript has improved in its revised version. However, some important methodological aspects were not addressed by the authors in their revision, and results presentation needs further revision to allow a correct interpretation. Because of these limitations, the conclusions of the study and its significance seem limited.

**Response**: Thank you for the careful and thorough review and constructive comments on the first and second version, which have helped to significantly improve the manuscript. We very much appreciate your time and commitment to improve the paper. We hope that your comments have been addressed in this revised version and we look forward to your feedback.
